# Impact of Obesity on Outcomes Associated With Acute Alcoholic Pancreatitis

**DOI:** 10.7759/cureus.51653

**Published:** 2024-01-04

**Authors:** James Pellegrini, Andrej M Sodoma, Rezwan Munshi, Jose R Russe-Russe, Jonathan Arias, Kristen L Farraj, Richard G Pellegrini, Jaspreet Singh

**Affiliations:** 1 Gastroenterology, Nassau University Medical Center, East Meadow, USA; 2 Internal Medicine, South Shore University Hospital, Bay Shore, USA; 3 Internal Medicine, Nassau University Medical Center, East Meadow, USA; 4 School of Medicine, St. George's University, St. George's, GRD; 5 Gastroenterology, South Shore University Hospital, Bay Shore, USA

**Keywords:** average length of hospital stay, alcohol induced pancreatitis, alcohol induced, acute pancreatitis, obesity-related illnesses

## Abstract

The incidence and prevalence of obesity have been rising in the United States, negatively impacting the population's overall health. This study seeks to better understand the impact of obesity on patients presenting with acute alcoholic pancreatitis (AAP). Data collected using the National Inpatient Sample (NIS) from the fourth quarter of 2015 to 2019 with a principal diagnosis of AAP and secondary obesity were analyzed. Confounders were adjusted for multivariate regression analysis using a multitude of factors. A total of 229,510 patients were identified with a diagnosis of AAP, among which 14,150 were also identified as obese. A majority of the sample, both obese and non-obese patients with AAP, were middle-aged white females. The average comorbidity index (CCI) was lower in the non-obese cohort compared to the obese cohort. Compared to non-obese patients, patients with AAP who were obese had higher hospital charges and a longer LOS (p<0.05. Additionally, compared to non-obese patients, obese patients with AAP had higher odds of mortality and adverse events, such as acute renal failure and respiratory failure (p<0.05). Current research supports these complications, which have shown an association with increased visceral fat in or around the pancreas that can ultimately worsen acute pancreatitis outcomes and aggravate AAP by damaging the intestinal mucosal barrier. These findings should be considered when treating obese patients who develop AAP. Strategies to increase surveillance of such patients should be implemented to reduce complications and mortality in this population.

## Introduction

Acute alcoholic pancreatitis (AAP) is an inflammatory disease of the pancreas caused by excessive alcohol use. As the second most common cause of acute pancreatitis after gallstones, excessive alcohol consumption, defined as, on average, more than 48 grams of pure ethanol or about two standard drinks per day, is a leading contributor to gastrointestinal-related disease, of which acute pancreatitis is the single most common cause of hospitalization in the United States [1,2]. Complications can occur locally with necrosis of the pancreatic tissue or the development of a pancreatic abscess. Furthermore, systemic complications such as sepsis, shock, multi-organ failure, and acute respiratory distress syndrome commonly occur. These complications are associated with significant morbidity and mortality.

There are multiple contributory factors to AAP, but one that is rising in prevalence and incidence in the United States and negatively impacting overall health is obesity [3,4]. Obesity is a known predisposing factor to dyslipidemia and diabetes, both of which aid in the progression of pancreatitis. However, the direct measure of obesity’s effects on the severity of AAP requires further research. This study seeks to better understand the impact of obesity on patients presenting with AAP, with hopes of raising awareness of complications and improving mortality in patients admitted for AAP.

## Materials and methods

Data source

Our study is a retrospective cohort study using the combined fourth quarter of the 2015-2019 National Inpatient Sample (NIS), an initiative provided by the Healthcare Cost and Utilization Project (HCUP). The NIS is one of the largest databases available in the United States and is maintained by the Agency for Healthcare Research and Quality (AHRQ). It comprises over seven million unweighted and over 35 million weighted hospital encounters each year. The data provided in the database is initially unweighted; then, using an algorithm provided by HCUP, it is converted to weighted data, which allows for estimates on a national level. The Institutional Review Board (IRB) was not required for this study as the NIS includes patient information that has been de-identified and made publicly available.

Study population

The NIS includes a 20% random sample of all inpatient hospitalizations from over 45 states and contains one primary diagnosis and up to 39 secondary diagnoses using the ICD-10-CM codes (International Classification of Disease-10-clinical modification). ICD codes were used to identify patients with a principal diagnosis of AAP and a secondary diagnosis of obesity (BMI 30-40 kg/m2). In addition, patients with a new or prior diagnosis of AAP (ICD-10 code: K85.2) were identified as the subject population, and those who were not obese with AAP were the control population. Our primary endpoints were the odds of adverse outcomes such as acute renal failure, acute respiratory failure, and all-cause mortality.

Statistical analysis

The Pearson chi-square test and Student’s t-test were utilized for assessing categorical and continuous variables, respectively. After adjusting for baseline characteristics and comorbidities to account for confounding variables (Table 1), a two-step hierarchical multivariate regression model was used, emphasizing variables with p<0.05. These variables included age, gender, race, hospital bed size, the Charlson Comorbidity Index (CCI), type of insurance, median income based on zip code, day of admission, and hospital region. Stata Version 17 by StataCorp LLC (College Station, TX) was utilized for all statistical analyses.

**Table 1:** Baseline characteristics of acute alcoholic pancreatitis patients with and without obesity

Variables	With Obesity (14,150; 6.17%)	Without Obesity (215,360; 93.83%)	P-value
Age (mean, SD)	45.76 +/- 12.15	45.41 +/- 12.48	<0.0001
Charlson Comorbidity Index (mean, SD)	1.24 +/- 1.32	0.94 +/- 1.22	<0.0001
Sex (N, %)			<0.0001
Female	9,200 (65.02%)	148,448 (68.93%)	
Male	4,950 (34.98%)	66,912 (31.07%)	
Race (N, %)			<0.0001
White	8,707 (61.53%)	131,112 (60.88%)	
Black	3,053 (21.58%)	50,997 (23.68%)	
Hispanic	1,763 (12.46%)	22,031 (10.23%)	
Other	627 (4.43%)	11,220 (5.21%)	

## Results

A total of 229,510 patients were identified with a diagnosis of AAP from the NIS, among which 14,150 (6.17%) had a diagnosis of obesity while 215,360 (93.83%) did not. The majority of the sample for both non-obese and obese patients with AAP was middle-aged (45.41 +/- 12.48 years old in non-obese vs. 45.76 +/- 12.15 years old in obese), white (60.88% in non-obese vs. 61.53% obese), and female (68.93% non-obese vs. 65.02% obese) (Table 1). Average CCI was lower in the non-obese cohort compared to the obese cohort (0.94 +/- 1.22 non-obese vs. 1.24 +/- 1.32 obese), p<0.001. Furthermore, compared to non-obese patients, patients with AAP who were obese had higher hospital charges [adjusted mean difference (aMD) $1,838] and a longer LOS (aMD 47.5 minutes); all p>0.05. In addition, compared to non-obese patients, obese patients with AAP had higher odds of mortality [adjusted odds ratio (aOR) 1.79 (1.05-1.31), p-value of 0.017] and adverse events such as acute renal failure (aOR 1.16 (1.03-1.31), p-value <0.0001) and respiratory failure (aOR 1.55 (1.29-1.88), p-value 0.032) (Table 2).

**Table 2:** Adverse events in alcoholic pancreatitis patients with obesity

Adverse Events	Odds Ratio (95% Confidence Interval)	P-value
Acute renal failure	1.16 (1.03 to 1.31)	0.017
Acute respiratory failure	1.55 (1.29 to 1.88)	<0.0001
All-cause mortality	1.79 (1.05 to 3.05)	0.032

## Discussion

Obesity is defined as excessive and abnormal accumulation of body fat, leading to increased morbidity, impaired quality of life, and higher rates of death driven by comorbidities [5,6]. The World Health Organization (WHO) defines obesity as a BMI ≥ 30kg/m2 [7,8]. Our study found that patients suffering from AAP and obesity had an increased chance of all-cause mortality, acute respiratory failure, and acute renal failure [9,10]. 

The pathophysiology between obesity and AAP is not entirely understood, though there are several associated etiologies that can potentially exacerbate pancreatic necrosis. Hypertriglyceridemia, diabetes, and alcoholism have been shown to aid in the progression of pancreatitis [7]. There is extensive evidence that obesity can lead to dyslipidemia and diabetes [11,12]. Obesity increases serum triglycerides by increasing the hepatic production of VLDL particles and decreasing the metabolism of triglyceride-rich lipoproteins [11]. It is essential to address these risk factors when evaluating a patient suffering from AAP.

AAP in patients with obesity has been demonstrated to have higher mortality and in-hospital complications. A study by the Journal of the Endocrine Society found that morbidly obese patients (BMI≥ 40kg/m2) had increased odds of mortality, hypocalcemia, sepsis, and acute kidney injury [13]. In our study, we found an increase in all-cause mortality, acute respiratory failure, and acute renal failure in patients suffering from AAP and obesity (Table 2). Severe pancreatitis is associated with organ failure that fails to resolve in 48 hours, such as shock, pulmonary insufficiency, renal failure, or gastrointestinal bleeding [14]. The severity of acute pancreatitis can be worsened by obesity due to an increase in visceral fat around the pancreas [7]. Also, obesity is associated with a low-grade inflammatory state, which may predispose to the development of organ dysfunction in acute pancreatitis [14]. A combination of these factors likely leads to increased morbidity and mortality in obese patients within our study. Therefore, it is important to address obesity and associated risk factors when treating a patient with AAP.

Obesity is linked to increased levels of inflammatory mediators and proteins. Excess macronutrients within adipose tissue stimulate the release of tumor necrosis factor-α (TNF-α) and interleukin-6 (IL-6), leading to the synthesis and secretion of C-reactive protein (CRP) [15]. These increased inflammatory markers may be risk factors for cardiovascular diseases, atherosclerosis, metabolic syndrome, and other diseases such as AAP. Though amylase is the most commonly used biomarker for the diagnosis of acute pancreatitis, several cytokines have been implicated as indicators for organ failure in acute pancreatitis [16,17]. In a study by Malmstrom et al., TNF-α, IL-6, and interleukin-8 (IL-8) were found to be significantly elevated in AAP patients who developed renal, respiratory, and circulatory failure [16].

Severe AAP has been associated with multiorgan failure, with acute kidney injury being a frequent complication observed late in the course of the disease [18]. Though the pathophysiology is not completely understood, studies have shown that the release of proteases and cytokines leads to increased vascular permeability and hypoxemia, inflammation, vasoconstriction, intravascular coagulation, and direct nephrotoxic effects resulting in acute kidney injury [17,18]. Furthermore, in a study by Levy et al., hypovolemia caused by acute pancreatitis led to a decrease in glomerular filtration rate by 40% and plasma volume by 26% within four hours in dogs [19]. Current data has suggested that obesity is a risk factor for acute kidney injury [20]. A recent study concluded that for each 5 kg/m2 increase in BMI, there was a 10% increased risk of acute kidney injury and worse short- and long-term survival [20]. Therefore, it is no surprise that we observed a higher incidence of acute renal failure among patients with AAP and obesity compared to AAP without obesity.

Acute respiratory failure occurs when arterial blood is inadequately oxygenated by the lungs, leading to hypoxia and even death [21]. Several theories have focused on the effects of obesity on acute pancreatitis, causing acute respiratory failure. Obesity can lead to increased visceral fat, which decreases diaphragmatic movement and inspiratory capacity. During times of distress, there is increased physiologic pulmonary arteriovenous shunting and hypoxemia [22]. In a study by Porter et al., obesity was observed to be an independent predictor of respiratory failure in severe acute pancreatitis [23]. A study by Lankisch et al. further concluded that obesity increases the incidence of shock, renal insufficiency, and respiratory insufficiency, necessitating artificial ventilation [24,25]. Though the link between obesity, AAP, and acute respiratory failure is not completely understood, our study adds to the body of research showing a positive correlation.

Limitations to this study include diagnostic coding errors, providers inaccurately documenting obesity, the nature of the dataset, and the possibility of inaccurate calculation of the disease burden due to multiple readmissions of the same patient. Furthermore, it is difficult to assess the nature and severity of AAP as it is limited by ICD-10 coding, and therefore, the adjusted model could not take into consideration the AAP disease severity. A future study with variables that can account for AAP severity would further elaborate on the findings of this paper. Confounders were adjusted for multivariate regression analysis using age, gender, race, hospital bed size, CCI, type of insurance, median income based on zip code, day of admission, and hospital region. Certain information was not available to us, such as smoking status, laboratory values, imaging, and medication use.

## Conclusions

Hypertriglyceridemia, diabetes, and alcoholism have been shown to complicate the progression of pancreatitis. Additionally, obesity can further complicate the multisystem clinical course of various diseases. Our study confirms these findings, as the average baseline comorbidity was lower in non-obese patients. It has been established that patients with a history of alcoholism are more likely to develop hypertriglyceridemia and diabetes. Furthermore, increasing visceral fat in or around the pancreas has been linked to worsening acute pancreatitis outcomes and aggravation of AAP by disrupting the intestinal mucosal barrier, according to current research. As a result, our findings cumulatively show that obese patients with AAP have a higher risk of in-hospital mortality. This finding indicates the need for closer surveillance of obese patients with AAP in hospitals, as they may be more susceptible to complications and mortality.
